# The Influence of ACE Insertion/Deletion Gene Polymorphism on the Risk of IgA Nephropathy: A Debatable Topic

**DOI:** 10.1155/2021/3112123

**Published:** 2021-11-18

**Authors:** Fen-Fen Chu, Shi-Kun Yang, Wen-Li Zeng

**Affiliations:** ^1^Department of Nephrology, The First Affiliated Hospital of the University of South China, Hengyang 421001, Hunan Province, China; ^2^Department of Nephrology, The Third Xiangya Hospital of Central South University, Changsha 410013, Hunan Province, China

## Abstract

**Background:**

The connection between angiotensin-converting enzyme insertion/deletion (ACE I/*D*) gene polymorphisms and IgA nephropathy (IgAN) was conflicting. This pooled analysis was performed to explore this issue.

**Methods:**

All eligible investigations were identified from various electronic databases, and the pooled analysis was evaluated using Stata software.

**Results:**

27 studies with 2538 IgAN cases and 3592 controls were included. In overall subjects, ACE *D* allele, DD, and II genotype were associated with IgAN susceptibility (D vs. I: OR = 1.21, 95% CI: 1.10–1.32, *P* < 0.001; DD vs. ID + II: OR = 1.38, 95% CI: 1.20–1.60, *P* < 0.001; and II vs. DD + ID: OR = 0.83, 95% CI: 0.73–0.95, *P*=0.007). In Asian and Chinese patients, ACE I/*D* gene polymorphism was also correlated with IgAN vulnerability. Moreover, ACE *D* allele, DD, and II genotype were correlated with the progression of IgAN (D vs. I: OR = 1.37, 95% CI: 1.09–1.73, *P*=0.008; DD vs. ID + II: OR = 1.57, 95% CI: 1.06–2.31, *P*=0.024; and II vs. DD + ID: OR = 0.69, 95% CI: 0.49–0.99, *P*=0.045). Conversely, in Caucasian subjects, there was no link between ACE I/*D* gene polymorphism and the risk of IgAN.

**Conclusion:**

ACE I/*D* gene polymorphism was correlated with the vulnerability and progression of IgAN in Asian and Chinese patients, and ACE *D* allele and DD homozygote genotype could be adverse factors for IgAN, while the II homozygote genotype could be an advantage factor. But, no significant association was found between ACE I/*D* gene polymorphism and IgAN in Caucasians.

## 1. Introduction

IgA nephropathy (IgAN) is a common type of glomerulonephritis globally; in the Pacific Asian region, the prevalence rate of IgAN is even more high [1]. It is a serious public health problem with very high mortality and morbidity, and recent studies have showed that approximately 20% IgAN patients would progress to end-stage renal disease (ESRD) if lacking effective treatment [2]. The presence of excessive immune complexes including IgA in the glomerulus was a key mechanism of IgAN. The current therapy was mainly on corticosteroids and immunosuppressors [3]. However, these measures were not fully effective. Recent research found that susceptibility to IgAN was influenced by a confluence of some genetic factors, and more importantly, some single-gene polymorphisms were associated with the susceptibility of IgAN [4].

Angiotensin-converting enzyme (ACE) was a key enzyme that converted angiotensin I to angiotensin II. The ACE gene consisted of 26 exons and located on chromosome 17q23 [5]. Single-nucleotide polymorphisms (SNPs) frequently occurred in the ACE gene; in the 16th intron of the ACE gene, a 287 bp fragment recognized as insertion/deletion (I/*D*) polymorphism has been found, and based on this I/*D* polymorphic marker locus, three genotypes could be defined: DD, II homozygotes, and ID heterozygote [6]. It has been found that serum ACE activity could be affected by ACE I/*D* gene polymorphisms, and these subjects with the *D* allele always have higher level of ACE activity [7]. Some research studies have demonstrated that ACE I/*D* polymorphism would affect the vulnerability of IgAN [8–10]. However, up to now, the role of ACE I/*D* polymorphism in the occurrence of IgAN is still inconsistent and controversial. So, in this study, we included much more high-quality studies to further assess the influence of ACE I/*D* gene polymorphism on IgAN susceptibility.

## 2. Methods

### 2.1. Study Search and Inclusion Strategy

We searched relevant studies from various electronic databases (e.g., PubMed, Scopus, and CNKI database) for eligible trials (all till Oct 2021). Multiple search keywords were used: IgA nephropathy, renal disease, kidney, nephropathy, ACE, ACE I/*D*, angiotensin-converting enzyme, insertion/deletion, gene, and gene polymorphism. The inclusion criteria of this study included the following: (1) the study included two comparison groups (IgA patients vs. control subjects or progression IgA patients vs. nonprogression IgA patients). (2) The number of ACE I/*D* genotypes could to be calculated. (3) The genotype distributions in control group was in accordance with Hardy–Weinberg Equilibrium (HWE).

### 2.2. Data Analysis

In this study, we used the Stata software to perform this pooled analysis. An odds ratio (OR) was calculated to evaluate the influence of ACE I/*D* gene polymorphism on IgAN susceptibility, and four different genetic models were used: model 1, allele *D* vs. allele I; model 2, genotype DD vs. genotype ID + II; model 3, genotype II vs. genotype DD + ID; and model 4, genotype ID vs. genotype DD + II. When a *P* value was less than 0.05, it was considered as statistically significant for the pooled OR. The heterogeneity among various included studies was evaluated using a *Q* test. Additionally, Begg' *s* test was completed to evaluate the publication bias; when the *P* value of Begg' *s* test was less than 0.05, potential publication bias was most likely to exist [11].

## 3. Results

### 3.1. Characteristics of the Included Studies

As shown in Table 1, 27 studies were finally included in our study [9,12–37], but 2 trials were excluded due to the fail to meet the HWE [32,35]. The main features of the included trials are described in Table 1. 6130 participants with 2538 cases and 3592 controls were included, and 9 of the studies were written in Chinese and 18 in English, from a total of 11 countries. The average age of participants ranged from 6 to 83 years. Additionally, the number of various genotypes were extracted and shown in Table 1.

### 3.2. The Link between ACE I/*D* Gene Polymorphism and IgAN Risk

**Figure 1:**
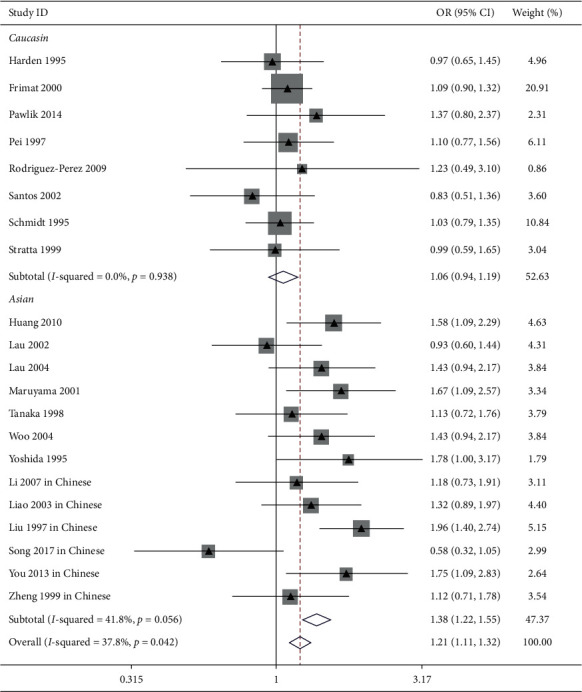
Forest plot of ACE I/*D* gene polymorphism linked with IgAN susceptibility (D allele vs. I allele). Pooled analysis indicated that the ACE gene locus D allele was a risk factor of IgAN in the overall and Asian subjects.

**Figure 2:**
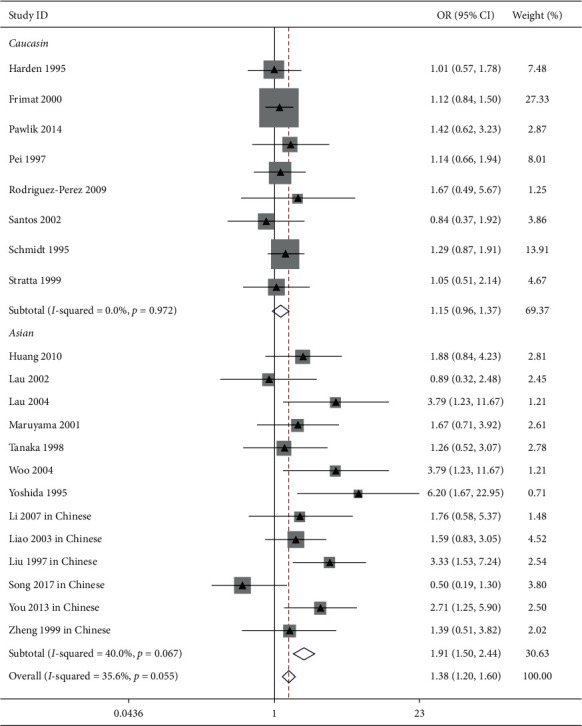
Forest plot about the link between ACE I/*D* gene polymorphism and IgAN risk (genotype DD vs. genotype ID + II). It showed that the ACE gene locus DD genotype was a risk factor of IgAN in the overall and Asian patients.

**Figure 3:**
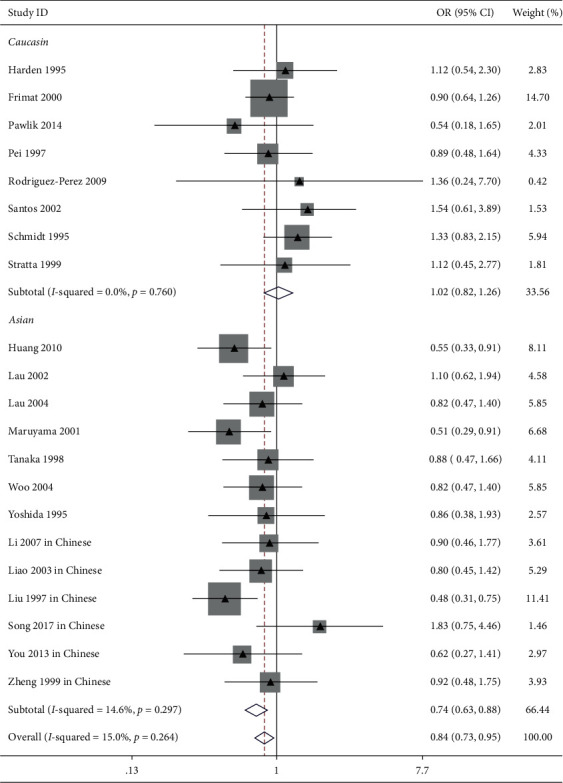
Forest plot about the link between ACE I/*D* gene polymorphism and IgAN risk (II genotype vs. DD + ID genotype). Evaluates of pooled OR for IgAN showed that the ACE gene locus II genotype was a protective factor of IgAN in the overall and Asian populations.

The influence of ACE I/*D* gene polymorphism on IgAN risk was reported in 21 trials [9,12,13,15,23,26,28,30,31,33,34,36,37]. Our pooled analysis showed that ACE I/*D* gene polymorphism was associated with IgAN risk in the general populations (allele *D* vs. allele I: OR = 1.21, 95% CI: 1.10–1.32, *P* < 0.001, Figure 1; genotype DD vs. genotype ID + II: OR = 1.38, 95% CI: 1.20–1.60, *P* < 0.001, Figure 2; and genotype II vs. genotype DD + ID: OR = 0.83, 95% CI: 0.73–0.95, *P*=0.007, Figure 3 and Table 2).

In the Asian patients, we also found a clear correlation between allele D/genotype DD and IgAN susceptibility (allele *D* vs. allele I: OR = 1.38, 95% CI: 1.22–1.55, *P* < 0.001, Figure 1; genotype DD vs. genotype ID + II: OR = 1.91, 95% CI: 1.50–2.44, *P* < 0.001, Figure 2). Conversely, our analysis indicated that the ACE II genotype was a protecting factor against IgAN (genotype II vs. genotype DD + ID: OR = 0.74, 95% CI: 0.63–0.88, *P* < 0.001, Figure 3 and Table 2).

**Figure 4:**
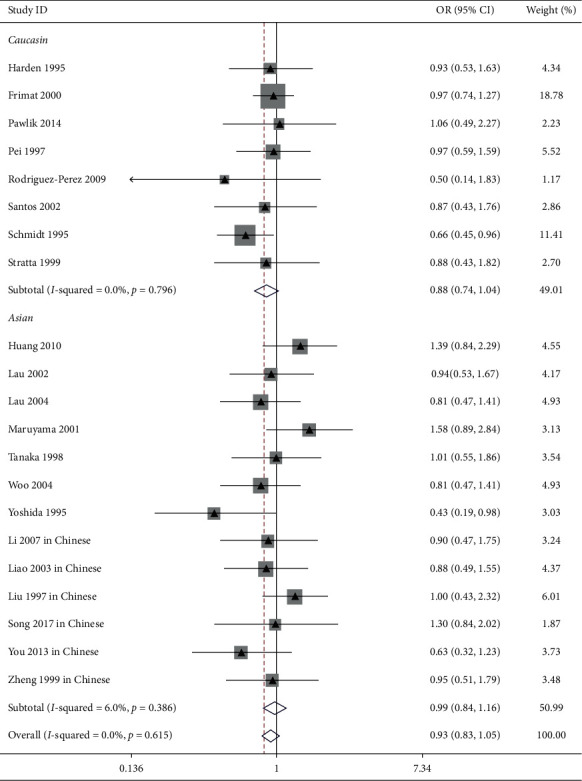
Forest plot about the link between ACE I/*D* gene polymorphism and IgAN susceptibility (genotype ID vs. genotype DD + II). Evaluates of pooled OR for IgAN showed that the ACE gene locus ID genotype was not associated with IgAN.

In Caucasian subjects, the pooled analysis including 8 trials showed that there was no obvious link between ACE I/*D* gene polymorphism and IgAN risk (allele *D* vs. allele I: OR = 1.06, 95% CI: 0.94–1.19, *P*=0.369, Figure 1; genotype DD vs. genotype ID + II: OR = 1.15, 95% CI: 0.96–1.37, *P*=0.137, Figure 2; genotype II vs. genotype DD + ID: OR = 1.02, 95% CI: 0.82–1.26, *P*=0.865, Figure 3; and genotype ID vs. genotype DD + II: OR = 0.88, 95% CI: 0.74–1.04, *P*=0.125, Figure 4, Table 2).

In this study, 8 trials reported the influence of ACE I/*D* gene polymorphism on IgAN vulnerability in Chinese patients. In line with Asian subjects, the pooled analysis indicated that allele *D* and genotype DD were risk factors for IgAN (allele *D* vs. allele I: OR = 1.41, 95% CI: 1.21–1.64, *P* < 0.001, Figure 1; genotype DD vs. genotype ID + II: OR = 1.89, 95% CI: 1.41–2.55, *P* < 0.001, Figure 2). Conversely, the ACE II genotype was an advantage factor for IgAN patients (genotype II vs. genotype DD + ID: OR = 0.71, 95% CI: 0.58–0.88, *P*=0.001, Figure 3 and Table 2).

### 3.3. The Link between ACE I/*D* Gene Polymorphism and IgAN Progression

There were 6 studies evaluating the influence of ACE I/*D* gene polymorphism on the progression of IgAN [14,23,25,28,29], and the pooled analysis found that ACE I/*D* gene polymorphism was associated with IgAN progression (allele *D* vs. allele I: OR = 1.37, 95% CI: 1.09–1.73, *P*=0.008; genotype DD vs. genotype ID + II: OR = 1.57, 95% CI: 1.06–2.31, *P*=0.024; and genotype II vs. genotype DD + ID: OR = 0.69, 95% CI: 0.49–0.99, *P*=0.045, Table 2).

### 3.4. Publication Bias

**Figure 5:**
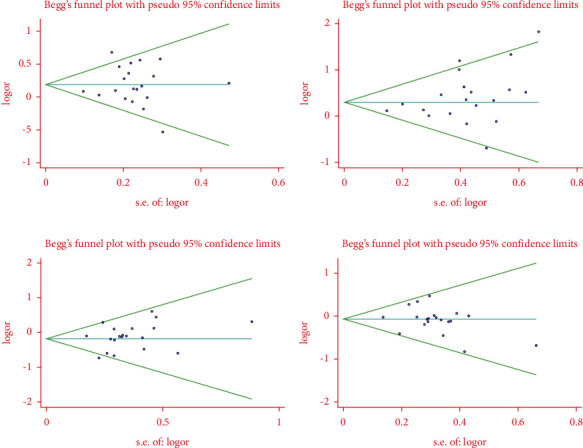
The funnel plots of different pooled analysis models. (a) Allele D vs. allele I; (b) genotype DD vs. genotype ID + II; (c) genotype II vs. genotype DD + ID; and (d) genotype ID vs. genotype DD + II.

The publication bias has been assessed using Begg' *s* test and Funnel plots. Potential publication bias was found in the analysis for the link of genotype DD with IgAN risk (DD vs. ID + II genotype: Begg' *sP*=0.044). Additionally, the funnel plot was asymmetrical (Table 2 and Figure 5).

## 4. Discussion

Our study demonstrated that the ACE I/*D* polymorphism was associated with the susceptibility and progression of IgAN. Specifically, it showed that the ACE I/*D* gene *D* allele and DD genotype were risk factors for IgAN, while the II genotype was an advantageous factor for IgAN.

This study was not the first pooled analysis to explore the relationship between ACE I/*D* polymorphism and IgAN; a small amount of previous meta-analysis about this controversial issue has been performed. In 2001 and 2006, two meta-analyses including only 5 reports have been completed [38,39]; these two research studies found that the association between the genetic susceptibility of IgAN and ACE I/*D* gene polymorphism was not significant. However, another pooled analysis performed by Yong showed that the ACE I/*D* polymorphism DD genotype was associated with IgAN progression both in Asian and Caucasians patients and the DD genotype was associated with IgAN risk in Asians [40]. As the number of included studies in these pooled analyses was small, in order to gain a more credible result, we reexamined the related trials and included 21 studies. We found that the allele *D* and genotype DD were correlated with IgAN risk and progression only in Asian patients. Similarly, Qin et al. found that the allele *D* and genotype DD were associated with IgAN susceptibility only in Asians, but there was no significant association between the allele *D* or genotype DD and the progression of IgAN [10]. Due to the fact that we included much more trials in our analysis compared to the previous research, we believed that our findings were more credible.

IgAN is a common type of glomerulonephritis globally, and it is a major cause of chronic renal failure among East Asian countries. The pathogenesis of IgAN is extremely complex, and immunological renal injury mediated by IgA is traditionally associated with IgAN [1,2]. It has been demonstrated that IgA immune complexes depositing in the glomerular mesangium could activate inflammatory response, profibrotic cell proliferation, and even the formation of a glomerular crescent [3]. But, more and more research suggested that renin-angiotensin-aldosterone system (RAAS) activation played a vital role in the development and occurrence of IgAN. Angiotensin II (AT II) was a pivotal factor of RAAS, and growing evidence indicated that AT II was a potential proinflammatory mediator associated with the renal tubule interstitial fibrosis [41]. The activation of AT II resulted in the production of various inflammatory cytokines, such as TNF-a, IL-6, IL-8, and TGF-*β* [42]. Consequently, these cytokines could greatly promote the occurrence and development of IgAN.

Recently, the researchers found that IgAN was also influenced by a confluence of environmental and genetic factors. Familial aggregation of IgAN indicated that genetic factor might participate in the pathogenesis of IgN [43]. With the technological development of genetic studies, especially the Genome Wide Association Study widely used, several SNPs within immune- or hypertension-related genes have been found significantly associated with IgAN. ACE was a pivotal factor of RAAS, the ACE gene contained 26 exons and 25 introns locating on 17q23 [6], and some polymorphic genetic markers of ACE gene have been found. Among these polymorphic markers, insertion or deletion (I/*D*) polymorphism (rs4340) of a 287 bp Alu in the 16th intron was the most investigated [6]. As mentioned earlier, there was a close relationship between the occurrence of IgAN and RAAS activation, and ACE played a vital role in the activation of angiotensin II. It was worth noting that the ACE level was strongly linked with ACE I/*D* polymorphism, and in those patients with the ACE DD genotype, the level of ACE expression and activity both in renal tissue and in serum were all markedly increased [44,45]. The insertion or deletion of base in the ACE gene might alter the expression or stabilization of ACE via influencing the stability of ACE mRNA. Thus, it could be seen that ACE *D* allele carriers had higher ACE level and consequently possessed higher risk to IgAN vulnerability.

In this study, the pooled analysis results indicated that the relationship between I/*D* polymorphism of ACE gene locus and IgAN risk was inconsistent in different races.

In Asian and Chinese patients, we found that the genotype DD, II, and allele *D* were associated with the vulnerability and progression of IgAN, but there was no evident correlation in Caucasian patients. This might be due to the differences in the environment and genetic backgrounds. Also, ACE inhibitors could affect the analysis results, while some studies in Chinese subjects did not clearly state whether to exclude those patients using ACE inhibitors. In short, the understanding about the ACE I/*D* genetic susceptibility difference was acquainted scarcely, and future research is needed to explore the specific mechanisms.

Some potential limitations should be discussed in our study. Firstly, the sample sizes in some included trials were small. Secondly, public bias was existed in this meta-analysis. Third, the included trials were from various countries, but the trials were published only in English and Chinese, which would result in reporting bias. Fourth, some included studies did not clearly state whether to exclude those subjects using ACE inhibitors. Finally, our study was mainly focused on ACE I/*D* genetic alteration, and the relationship between many other genes SNPs and IgAN susceptibility needed to be further explored.

## 5. Conclusions

Our pooled analysis supported that ACE gene locus I/*D* polymorphisms were linked with the susceptibility and progression of IgAN in Asian and Chinese individuals. ACE gene locus allele *D* and genotype DD could be risk factors of IgAN vulnerability. Conversely, those subjects carrying the ACE II genotype would reduce the risk of IgAN susceptibility. However, no obviously correlation was found between ACE I/*D* gene polymorphism and IgAN in Caucasian patients. In future studies, much more high-quality trials are required to clarify it.

## Figures and Tables

**Table 1:** Characteristics of studies included in the meta-analysis.

Trials	Design	Country; ethnicity	Year	Sex (M/F)	Case	Control	Case			Control			HWE (p)
							DD	ID	II	DD	ID	II	
Harden, 1995	Case control	UK; European	NR	NR	`100	98	40	41	19	39	42	17	0.3351
Huang, 2010	Case control	China; Asian	NR	NR	130	120	19	67	44	10	52	58	0.7280
Hunley, 1996	Case control	USA; American	6–83	43/21	40^#^	24^∗^	8	24	8	1	14	9	0.1258
Lau, 2002	Case control	Singapore; Asian	NR	45/55	100	90	8	43	49	8	40	42	0.7265
Lau, 2004	Case control	China; Asian	P: 43 ± 10; C: 48 ± 14	P: 56/62; C: 42/52	118	94	17	48	53	4	43	47	0.1280
Frimat, 2000	Case control	France; European	NR	NR	274	960	89	132	53	288	470	202	0.6891
Maruyama, 2001	Case control	Japan; Asian	P: 10.6 ± 2.9; C : NR	P: 56/39; C : NR	95	99	15	42	38	10	33	56	0.1362
Pawlik, 2014	Case control	Poznan; European	P : NR; C: 38.1 ± 12.8	P : NR; C: 105/82	31	187	10	17	4	47	100	40	0.3315
Pei, 1997	Case control	Canada; American	NR	NR	168	100	55	81	32	30	49	21	0.9045
Rodríguez-Pérez, 2009	Case control	Spain; Europen	P: 33–46; C: 29–46	P: 10/3; C: 42/9	13	51	7	4	2	21	24	6	0.8287
Santos, 2002	Case control	USA; American	NR	NR	79	53	17	45	17	13	32	8	0.1119
Schmidt, 1995	Case control	Germany, Australia, Italy; mixed	NR	P: 153/51; C : NR	204	234	79	81	44	77	117	40	0.6949
Stratta, 1999	Case control	Italy; European	NR	P: 67/14; C: 25/25	81	50	35 20^#^	30 16^#^	16 5^#^	21 9^∗^	20 13^∗^	9 8^∗^	0.2853 0.4684
Suzuki, 2000	Case control	Japan; Asian	P: 39.2 ± 12.3; C: 34.9 ± 13.5	P: 49/34; C: 117/133	83^#^	250^∗^	13^#^	31^#^	39^#^	35^∗^	107^∗^	108^∗^	0.3100
Syrjänen, 2000	Case control	Finland; European	NR	NR	26^#^	142^∗^	9^#^	16^#^	1^#^	45^∗^	67^∗^	30^∗^	0.5861
Tanaka, 1998	Case control	Japan; Asian	NR	NR	97	71	15	48	34	9	35	27	0.6513
Woo, 2004	Case control	Singapore; Asian	P: 43 ± 10; C: 48 ± 14	P: 56/62; C: 42/52	118	94	17	48	53	4	43	47	0.1280
Yoshida, 1995	Case control	Japan; Asian	P: 38.2 ± 2.1; C: 35.8 ± 1.8	P: 34/19; C: 24/22	53	46	16 12^#^	17 8^#^	20 8^#^	3 4^∗^	24 9^∗^	19 12^∗^	0.2045 0.3225
Hu, 1997	Case control	China; Asian	36.5 ± 7.5	55/27	51^#^	31^∗^	14^#^	26^#^	11^#^	8^∗^	13^∗^	10^∗^	0.3797
Li, 2007	Case control	China; Asian	P: 34.8 ± 15.8; C: 33.1 ± 13.9	P: 30/34; C: 26/54	64	80	8	32	24	6	42	32	0.1196
Liao, 2003	Case control	China; Asian	P: 17–68; C: 19–65	P: 60/35; C: 58/47	95	105	27	35	33	21	42	42	0.0877
Liu, 2005	Case control	China; Asian	P: 29.7 ± 10.7; C: NR	P: 98/48; C : NR	146	146	18	78	50	9	86	51	0.0006
Liu, 1997	Case control	China; Asian	P: 9–42; C: 18–55	P: 106/71; C: 54/96	177	150	31	81	65	9	59	82	0.7059
Song, 2017	Case control	China; Asian	P: 42.9 ± 12.3; C: 43.7 ± 13.5	P: 23/22; C: 27/18	45	45	9	18	18	15	18	12	0.1876
Xu, 2001	Case control	China; Asian	P: 11–56; C: 20–68	P: 63/47; C: 73/43	110	116	25	39	46	24	40	52	0.0039
You, 2013	Case control	China; Asian	P: 33.6 ± 12.5; C: 31.5 ± 10.8	P: 36/32; C: 34/36	68	70	26	30	12	13	39	18	0.3154
Zheng, 1999	Case control	China; Asian	P: 29.8; C: 33.1	P: 44/28; C: 16/70	72	86	9	36	27	8	44	34	0.2419

*Note.* P: IgA nephropathy subjects; C: control subjects; HB, hospital based; PB: population based; NR: not reported; PCR, polymerase chain reaction; RFLP, restriction fragment length polymorphism. ^#^Progression of IgAN; ^∗^nonprogression of IgAN.

**Table 2:** Meta-analysis of the link between ACE gene locus I/*D* polymorphisms and the susceptibility and progression of IgAN.

Genetic contrasts	Group and subgroups	Study number	*Q* test *p* value	Model selected	OR (95% CI)	*P* value	Begg's test
D versus I	Overall	21	0.042	Fixed	1.21 (1.10–1.32)	<0.001	0.729
	Asian	13	0.056	Fixed	1.38 (1.22–1.55)	<0.001	—
	Caucasian	8	0.938	Fixed	1.06 (0.94–1.19)	0.369	—
	Chinese	8	0.035	Fixed	1.41 (1.21–1.64)	<0.001	—
	Non-Chinese	13	0.529	Fixed	1.12 (1.01–1.24)	0.031	—
	IgAN progression	6	0.128	Fixed	1.37 (1.09–1.73)	0.008	—

DD versus ID + II	Overall	21	0.055	Fixed	1.38 (1.20–1.60)	<0.001	0.044
	Asian	13	0.067	Fixed	1.91 (1.50–2.44)	<0.001	—
	Caucasian	8	0.972	Fixed	1.15 (0.96–1.37)	0.137	—
	Chinese	8	0.089	Fixed	1.89 (1.41–2.55)	<0.001	—
	Non-Chinese	13	0.373	Fixed	1.25 (1.06–1.47)	0.009	—
	IgAN progression	6	0.340	Fixed	1.57 (1.06–2.31)	0.024	—

II versus DD + ID	Overall	21	0.264	Fixed	0.83 (0.73–0.95)	0.007	0.294
	Asian	13	0.297	Fixed	0.74 (0.63–0.88)	<0.001	—
	Caucasian	8	0.760	Fixed	1.02 (0.82–1.26)	0.865	—
	Chinese	8	0.193	Fixed	0.71 (0.58–0.88)	0.001	—
	Non-Chinese	13	0.640	Fixed	0.93 (0.78–1.09)	0.386	—
	IgAN progression	6	0.117	Fixed	0.69 (0.49–0.99)	0.045	—

ID versus DD + II	Overall	21	0.615	Fixed	0.93 (0.83–1.05)	0.235	0.389
	Asian	13	0.386	Fixed	0.98 (0.84–1.16)	0.858	—
	Caucasian	8	0.796	Fixed	0.88 (0.74–1.04)	0.125	—
	Chinese	8	0.568	Fixed	1.01 (0.82–1.24)	0.936	—
	Non-Chinese	13	0.533	Fixed	0.89 (0.78–1.03)	0.131	—
	IgAN progression	6	0.600	Fixed	1.00 (0.72–1.39)	0.997	—

## Data Availability

The data used to support the findings of this study are available from the first author and corresponding author upon request. Since this is a pooled analysis, data from previous published studies were used.

## Abbreviations

SNPs: Single-nucleotide polymorphisms
HWE: Hardy–Weinberg equilibrium
OR: Odds ratio
CIs: Confidence intervals
